# The Neural Correlates of Belief Bias: Activation in Inferior Frontal Cortex Reflects Response Rate Differences

**DOI:** 10.3389/fnhum.2014.00862

**Published:** 2014-10-21

**Authors:** Caren M. Rotello, Evan Heit

**Affiliations:** ^1^Department of Psychological and Brain Sciences, University of Massachusetts, Amherst, MA, USA; ^2^School of Social Sciences, Humanities and Arts, University of California, Merced, CA, USA

**Keywords:** belief bias, reasoning, neuroscience, accuracy, congruency

The belief bias effect in reasoning (Evans et al., 1983) is the tendency for logical problems with believable conclusions (e.g., some addictive things are not cigarettes) to elicit more positive responses than those with unbelievable conclusions (some cigarettes are not addictive things). The effect of believability interacts with conclusion validity (see the lower rows of Table 1 for example data), leading many researchers to conclude that reasoning accuracy is greater for problems with unbelievable conclusions (e.g., Oakhill and Johnson-Laird, 1985; Newstead et al., 1992; Quayle and Ball, 2000). Dube et al. (2010, 2011) [see also Heit and Rotello (2014)] demonstrated that the typical ANOVA analysis of these behavioral data was inappropriate, and showed that a signal detection based interpretation of the data reached a different conclusion, namely that the effect of conclusion believability was to shift subjects’ response bias to be more liberal. Trippas et al. (2013) also concluded that conclusion believability consistently affected response bias, but that reasoning accuracy was additionally affected by believability under certain conditions (i.e., higher cognitive ability, complex syllogisms, unlimited decision time).

**Table 1:** **Data from Dube et al. (2010)**.

Experiment	Condition	Response rates				
		*H* = *P*(“valid”|	Miss = *P*(“invalid”|	*F* = *P*(“valid”|	CR = *P*(“invalid”|	Overall “valid”
		Valid)	Valid)	invalid)	invalid)	response rate
1	Liberal	0.79	0.21	0.67	0.33	0.730
	Conservative	0.55	0.45	0.31	0.69	0.430
2	Believable	0.86	0.14	0.61	0.39	0.735
	Unbelievable	0.68	0.32	0.32	0.68	0.500

The belief bias effect has also been studied in the neuroscience literature, although the focus has been slightly different. Whereas in the behavioral literature, researchers have focused on the accuracy with which subjects can discriminate valid from invalid conclusions, in the neuroscience literature, questions have centered on the brain regions responsible for resolving the conflict between the logically correct response to a problem and the believability of its conclusion. That is, neuroscience analyses have divided test trials into those for which validity and believability lead to the same conclusion (congruent trials) and those for which they lead to different conclusions (incongruent trials). A consistent finding is that the percentage of correct responses is higher for congruent than incongruent trials, an effect attributed to the competition between System 1, which drives belief-based responding, and System 2, which drives logic-based decisions (e.g., Goel et al., 2000; Tsujii and Watanabe, 2010; cf. Evans and Curtis-Holmes, 2005). A similarly consistent finding is the selective activation of right prefrontal cortex (rPFC) for incongruent, and not congruent, test trials, suggesting a role for rPFC in conflict detection and/or resolution (fMRI: Goel et al., 2000; Goel and Dolan, 2003; Stollstorff et al., 2012; fNIRS: Tsujii and Watanabe, 2009, 2010; Tsujii et al., 2010b; TMS: Tsujii et al., 2010a). For example, Stollstorff et al. (2012) noted that right lateral PFC “is consistently engaged to resolve conflict in deductive reasoning” (p. 28). In ERP, a late positivity for incongruent trials has been interpreted similarly (Luo et al., 2008, 2013). These data suggest that rPFC activation inhibits System 1 responding, a conclusion that is broadly consistent with the assumed inhibitory function of right inferior frontal cortex (Aron et al., 2014).

We will begin by showing that the partitioning of trials and subsequent analysis are based on faulty logic, such that the intended comparison of accuracy for congruent versus incongruent trials actually reflects differences in the “valid” response rates to believable and unbelievable problems. Using simple algebra, we show that accuracy for congruent and incongruent trials can only be equal when the ‘valid’ response rate does not vary with believability. Second, we will turn to the interpretation of the corresponding brain data, arguing that it is also flawed because of its dependence on those very same accuracy differences. Finally, we will suggest an alternative interpretation of rPFC activation in the belief bias task.

In belief bias studies, accuracy for the congruent trials, *A*_c_, is measured using percent correct. It is simply the average of the “valid” (hit) response rate in the believable condition (*H*_B_) and the “invalid” (correct rejection) response rate in the unbelievable condition (CR_U_):

(1) $$A_{C}=\frac{1}{2}\left(H_{B}+CR_{U}\right)$$

Likewise, accuracy for the incongruent trials, *A*_I_, is simply the average of the hit rate in the unbelievable condition and the correct rejection rate in the believable condition:

(2) $$A_{I}=\frac{1}{2}\left(H_{U}+CR_{B}\right)$$

For example, for the representative data in the lower rows of Table 1, *A*_C_ = 0.5(0.86 + 0.68) = 0.77, and *A*_I_ = 0.5 (0.68 + 0.39) = 0.54, implying that accuracy is higher for the congruent than the incongruent trials. Interestingly, the accuracy advantage seen for congruent trials is observed even though believability did not affect validity discrimination in this experiment (Dube et al., 2010, Exp. 2).

The interpretation of the neuroscience data on belief bias depends crucially on the difference in accuracy for congruent and incongruent trials. To understand these data, we first show that interpretation of the percent correct accuracy measure actually depends on response rate differences. Let us spend a moment examining how the accuracy difference could come about, by starting with the question of when accuracy for the two trial types would be equal. In other words, under what conditions does *A*_C_ = *A*_I_, or, equivalently, when is Eq. 3 true?

(3) $$\frac{1}{2}\left(H_{B}+CR_{U}\right)=\frac{1}{2}\left(H_{U}+CR_{B}\right)$$

Because the correct rejection rate, CR, equals 1 minus the false alarm rate, F, we can rewrite Eq. 3:

(4) $$\frac{1}{2}\left(H_{B}+1-F_{U}\right)=\frac{1}{2}\left(H_{U}+1-F_{B}\right)$$

Some reorganization and simplification yields

(5) $$\frac{1}{2}\left(H_{B}+F_{B}\right)=\frac{1}{2}\left(H_{U}+F_{U}\right)$$

Equation 5 is revealing, because the average of the hit and false alarm rates equals the “yes” rate (assuming equal number of target and lure trials). As Macmillan and Creelman (2005) showed, the yes rate is a measure of response bias, not accuracy. Thus, Eq. 5 shows that the congruent and incongruent trials can only yield equal accuracy (measured with percent correct; a related argument applies to *d*′) if the response rates to believable and unbelievable problems are the same. This bias restriction is unlikely to be met, because the belief bias effect itself is a difference in positive response rates with conclusion believability (e.g., Evans et al., 1983; Dube et al., 2010, 2011; Trippas et al., 2013). Believable problems tend to elicit more positive responses both for valid and invalid conclusions; thus, it is easy to see that the congruency analysis will produce *A*_C_ > *A*_I_. Starting with a version of Eq. 4 that assumes *A*_C_ > *A*_I_

(6) $$\frac{1}{2}\left(H_{B}+1-F_{U}\right)>\frac{1}{2}\left(H_{U}+1-F_{B}\right)$$

we can simplify and reorganize to see that *A*_C_ > *A*_I_ whenever

(7) $$H_{B}-H_{U}>F_{U}-F_{B}$$

Because both the hit and false alarm rate are higher to problems with believable conclusions, the left side of the inequality in Eq. 7 will be positive, and the right side will be negative: *A*_C_ will always be greater than *A*_I_ if believable conclusions elicit more positive responses than unbelievable conclusions. This observation generalizes to any empirical manipulation that elicits a response rate difference, as long as the more liberal condition is treated as analogous to the believable problems. For example, the upper rows of Table 1 show data from Dube et al. (2010) (Exp. 1), which was a syllogistic reasoning task on abstract problems that were structurally identical to those in their belief bias experiments. One group of subjects was told that 85% of the problems had a valid conclusion, and another group was told that 15% of the conclusions were valid, though in fact both groups were given identical problem sets in which 50% of conclusions were logically valid. Treating the liberal condition as analogous to the believable problems, and letting the conservative condition play the role of the unbelievable problems, we can compute *A*_C_ = 0.74 and *A*_I_ = 0.44, implying that accuracy is higher for the congruent than the incongruent trials despite the absence of any believable (or unbelievable) content.

We turn now to the neuroscience literature, for which we argue that differences in response rates have been misinterpreted as accuracy differences. Neuroscience studies of belief bias have consistently found selective activation of rPFC to incongruent trials (Goel et al., 2000; Goel and Dolan, 2003; Tsujii and Watanabe, 2009, 2010; Tsujii et al., 2010a,b; Stollstorff et al., 2012). Indeed, Tsujii and Watanabe (2009, 2010) and Tsujii et al. (2010b) took this general finding a step further. In each of these three studies, they reported a positive correlation between the magnitude of activation in rIFC and the difference in accuracy levels for incongruent and congruent trials. Tsujii and Watanabe (2009) wrote “subjects with enhanced activation in the right IFC could also perform better in conflicting [incongruent] reasoning trials” (p. 121). As we have seen, however, accuracy differences as a function of congruency simply reflect a different “valid” response rate to problems with believable and unbelievable conclusions. So, a better interpretation of these data is that right IFC activation correlates with the magnitude of that response rate difference. The scatter plots in each of these studies show that the highest degree of selective activation (largest difference for incongruent compared to congruent trials) corresponds to accuracy differences (incongruent minus congruent) that are zero or positive, meaning that those subjects showed an atypical response to the belief bias task: either they showed no response rate difference with believability (and thus had no accuracy difference, see Eq. 5) or they made more positive responses to unbelievable than believable conclusions (and thus had higher accuracy for incongruent trials than congruent, see Eq. 7).

Tsujii et al. (2010a) used TMS to show that disruption to right IFC increased the magnitude of the accuracy difference with congruency: subjects showed large accuracy advantages for congruent trials, which can only occur because of large response rate effects of believability (Eq. 7). Interestingly, disruption to left IFC eliminated the accuracy advantage for congruent trials, meaning that the “valid” response rate to believable and unbelievable conclusions was at least roughly equated (Eq. 5).

Our analysis of the accuracy effect of congruency shows that the analyses in the neuroscience literature on belief bias have not directly addressed why congruency differences occur, the brain regions responsible for conflict detection/resolution, or the relative involvement of reasoning Systems 1 (belief) and 2 (logic). None of those processes have been shown to be involved in the appearance of an accuracy difference with congruency (see Eqs 5 and 7). Instead, the selective activation of prefrontal cortex in response to incongruent problems must be a consequence of the response rate difference for believable and unbelievable problems.

The failure to consider response rate differences across conditions has also lead to the misinterpretation of behavioral data in a variety of domains (e.g., Verde and Rotello, 2003; Rotello et al., 2005; Dougal and Rotello, 2007; Evans et al., 2009; Mickes et al., 2012) and of other neuroscience data. For example, fMRI evidence from perceptual categorization and recognition tasks had been interpreted as showing distinct cortical systems for these tasks (e.g., Reber et al., 1998). However, Nosofsky et al. (2012) noted that the “yes” response rate also differs by task: categorization naturally suggests a more liberal response criterion than recognition. When activation patterns were compared for categorization tasks and a recognition task in which subjects were instructed to use a liberal recognition criterion, no differences in brain activation were found; the distinct patterns were attributable to the response bias difference.

Some recent neuroscience studies have explicitly manipulated the decision criterion across trials. In simple perceptual tasks such as line length discrimination, this can be accomplished by showing participants the length of the line to use as the boundary between “short” and “long” responses. Using this strategy, White et al. (2012) found left inferior temporal cortex, which is responsible for representing objects, was activated in response to the decision criterion itself. They suggested that the criterion value (here, an explicitly provided line length) was stored much like any other stimulus, and so its particular brain location would vary with the task. In the case of syllogistic reasoning, the decision criterion represents a level of evidence for the validity of the conclusion. Where this information would be stored is an interesting question to consider, but it seems that one possible place to starting looking would be in the right inferior frontal cortex. More generally, we see much promise in future neuroscience studies of belief bias that take account of what can be inferred from analysis of behavioral measures.

## Author Contributions

Caren M. Rotello identified the problem and wrote the first draft. Evan Heit provided critical revisions.

## Conflict of Interest Statement

The authors declare that the research was conducted in the absence of any commercial or financial relationships that could be construed as a potential conflict of interest.
